# A Rare Case of Erdheim-Chester Disease and Langerhans Cell Histiocytosis Overlap Syndrome

**DOI:** 10.1155/2015/949163

**Published:** 2015-10-22

**Authors:** Shahzaib Nabi, Adeel Arshad, Tarun Jain, Fawad Virk, Rohit Gulati, Rana Awdish

**Affiliations:** ^1^Department of Internal Medicine, Henry Ford Health System, 2799 W. Grand Boulevard, Detroit, MI 48202, USA; ^2^Department of Internal Medicine, Hamad Medical Corporation, Weill Cornell University, Doha, Qatar; ^3^Department of Pathology, Henry Ford Health System, 2799 W. Grand Boulevard, Detroit, MI 48202, USA; ^4^Department of Pulmonary and Critical Care Medicine, Henry Ford Health System, 2799 W. Grand Boulevard, Detroit, MI 48202, USA

## Abstract

A 48-year-old woman with a past medical history of seizures and end-stage renal disease secondary to obstructive uropathy from retroperitoneal fibrosis presented to the emergency department with seizures and altered mental status. A Glasgow Coma Scale of 4 prompted intubation, and she was subsequently admitted to the intensive care unit. Magnetic resonance imaging of the brain performed to elucidate the aetiology of her seizure showed a dural-based mass within the left temporoparietal lobe as well as mass lesions within the orbits. Further imaging showed extensive retroperitoneal fibrosis extending to the mediastinum with involvement of aorta and posterior pleural space. Imaging of the long bones showed bilateral sclerosis and cortical thickening of the diaphyses. Imaging of the maxillofacial structures showed osseous destructive lesions involving the mandible. These clinical and radiological features were consistent with a diagnosis of Erdheim-Chester disease; however, the patient's skin biopsy was consistent with Langerhans cell histiocytosis.

## 1. Introduction

Both Erdheim-Chester disease (ECD) and Langerhans cell histiocytosis (LCH) are histiocytic disorders and are extremely rare. Review of the literature shows cases of overlap between ECD and LCH. The purpose of writing this case report is to strengthen the limited knowledge of such an overlap syndrome. Moreover, such case reports suggest that these 2 disorders might not be completely separate entities and, hence, can lead to a better understanding of the pathophysiology of these disorders.

More importantly, such case reports aid clinicians in making an accurate diagnosis when faced with this rare group of disorders. Early diagnosis and prompt treatment can confer improved outcomes, both clinically and in terms of patient satisfaction.

## 2. Case Presentation

Our patient is a 48-year-old female with a past medical history of end-stage renal disease secondary to obstructive uropathy from retroperitoneal fibrosis (with bilateral ureteral stents). She also had a past medical history of intermittent seizures. She presented to the hospital with altered mental status that had been waxing and waning for the past 2 weeks.

On physical examination in the emergency department, she was found to have a temperature of 39.2°Celsius, pulse of 110 beats/minute, respiratory rate of 22 breaths/minute, and blood pressure of 83/42 mmHg. On neurological examination, the patient was found to be alert to person, but not to time or place. She was moving all 4 extremities without any apparent focal neurological deficit. Reflexes were normal in both upper and lower extremities. The rest of the neurological examination was limited as the patient was unable to follow commands. Cardiovascular examination showed normal S1 and S2 with no added sounds. Respiratory and abdominal examinations were unremarkable. Skin examination showed chronic ulcers on the lower extremities in different stages of healing. She was also found to have scaly erythematous papules in her right axillary skin fold.

Her family history was unremarkable. She was a lifelong nonsmoker and nonalcoholic and never used any illicit drugs. She was on levetiracetam for seizures, which she was taking as prescribed (according to the family).

Patient was found to be in septic shock secondary to infected ureteral stents. She was admitted to the intensive care unit and her ureteral stents were removed. She received broad spectrum intravenous antibiotics and aggressive fluid resuscitation with resolution of her septic shock and normalization of her hemodynamics. However, her mental status did not improve with resolution of the sepsis. Imaging studies showed a dural-based mass in the left temporoparietal region as well as mass lesions within the orbits. Further work-up showed lytic lesions involving the mandible and axilla. Bilateral cortical thickening and sclerosis of the long bones of the extremities was also noted. Her retroperitoneal fibrosis was found to be extending into her mediastinum with encasement of the vessels and pleural involvement. These findings were consistent with a diagnosis of ECD; however, the patient's skin biopsy was consistent with a diagnosis of LCH.

## 3. Investigations

### 3.1. Pertinent Imaging Investigations

**Figure 1:**
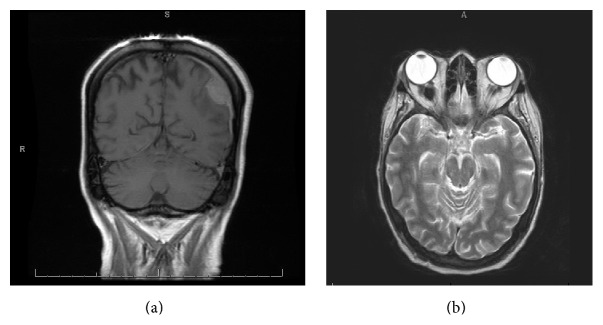
MRI brain coronal section (a) and transverse section (b) showing dural-based mass in the left temporoparietal region as well as mass lesions within the orbits.

**Figure 2:**
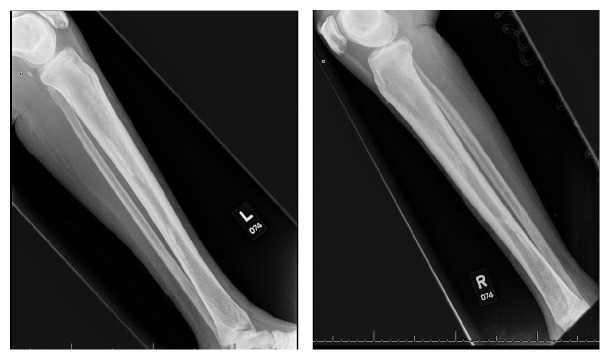
X-ray of both legs showing regions of sclerotic cortical thickening identified in the posterior distal metadiaphysis and anterior metadiaphysis of the tibia with additional sclerosis of the metadiaphyseal regions of the fibula.

Magnetic resonance imaging of the brain showed a dural-based mass within the left temporoparietal lobe. Evidence of mass lesions within the orbits with tram track appearance was found on fluid-attenuated inversion recovery imaging (Figure 1). X-rays of the long bones of the extremities displayed evidence of sclerosis and cortical thickening involving the bilateral radial and ulnar diaphyses. Regions of metadiaphyseal sclerosis and cortical thickening were identified in the bilateral tibias and fibulas (Figure 2).

**Figure 3:**
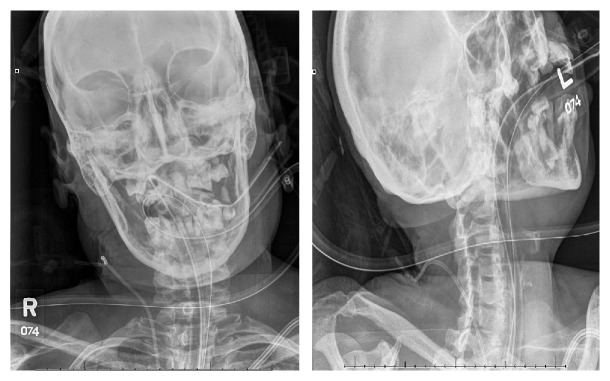
X-ray of the skull, showing lytic lesions in the mandible and bilateral maxillae.

**Figure 4:**
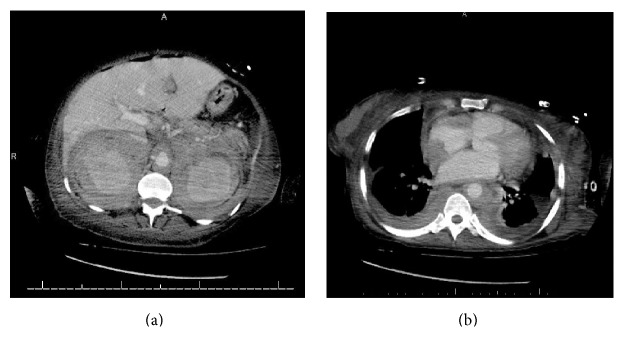
CT of the abdomen (a) showing extensive retroperitoneal soft tissue (fibrosis) surrounding the kidney and vessels. The soft tissue is seen extending into the mediastinum with involvement of the pleura, heart, and vessels (b).

Radiographic imaging of the skull showed large lytic lesions involving the alveolar regions of bilateral maxillae, as well as symphysis, body, and alveolar ridge of the mandible bilaterally (Figure 3). Extensive soft tissue surrounding both of the kidneys, the aorta, and the retroperitoneum, extending along the great peritoneal vessels, was found on computed tomography of the abdomen and pelvis. Computed tomography of the chest further showed that the soft tissue from the retroperitoneal region extended into the mediastinum and was surrounding the heart, the aorta, and pulmonary arteries and extending along the paraspinal region (Figure 4).

### 3.2. Pertinent Laboratory Investigations

Complete blood count (on presentation) showed a white blood cell count of 28,000/mm^3^ and haemoglobin of 7.7 gm/dL (anemia secondary to end-stage renal disease). Basic metabolic panel is as follows: normal electrolytes and creatinine of 4.4 mg/dL (due to end-stage renal disease). IgG 4 level was found to be 37 mg/dL. Triglycerides and fibrinogen level was within normal limits.

**Figure 5:**
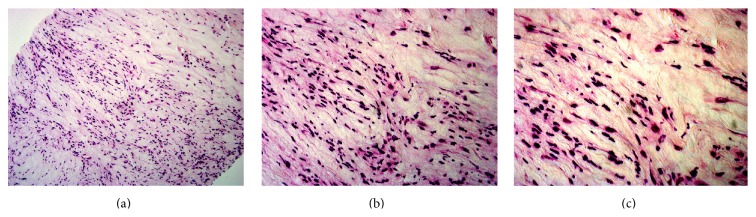
Retroperitoneal biopsy, H&E stain, ×200 (a), ×400 (b), and ×600 (c) showing fibrotic tissue and abundance of foamy macrophages.

**Figure 6:**
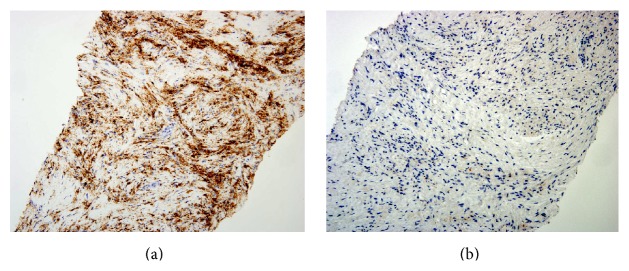
Retroperitoneal biopsy: immunohistochemical stain showing positivity for CD68 (a) and negativity for S100 (b).

Retroperitoneal/perirenal soft tissue biopsy showed evidence of fibrosis with abundance of foamy macrophages (Figure 5). CD68 immunostain was positive. Immunostains were negative for S100, CD1a, and cytokeratins (Figure 6). We were unable to assess the BRAF status as the biopsy specimen was insufficient to test for BRAF status.

**Figure 7:**
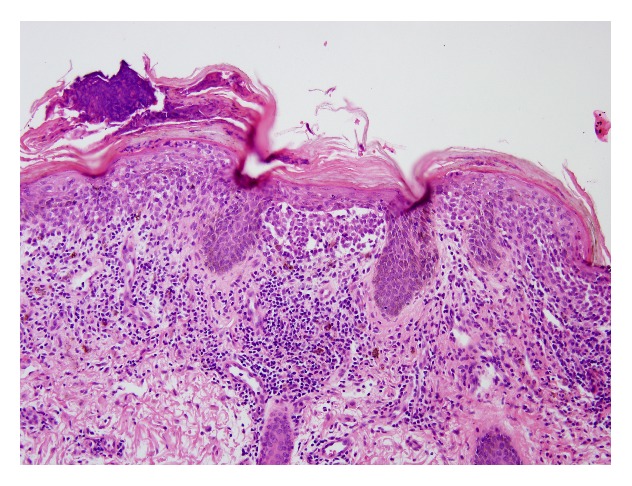
Skin biopsy, H&E stain (×200): dense deposit of Langerhans cells with eosinophilic cytoplasm and indented nuclei in the superficial dermis and epidermis highlights epidermotropism.

**Figure 8:**
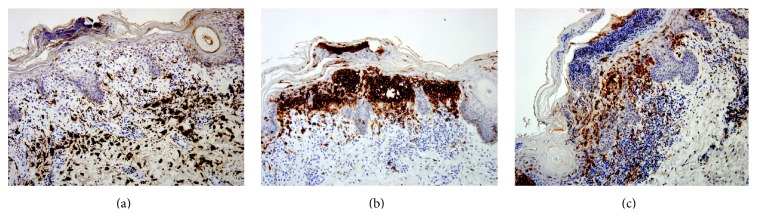
Skin biopsy: immunohistochemical stain showing positivity for CD68 (a), CD1a (b), and S100 (c).

Axillary skin punch biopsy showed numerous Langerhans' cells in the epidermis and superficial dermis (Figure 7). Immunostains were positive for CD68, S100, and CD1a (Figure 8). These findings were consistent with a diagnosis of LCH.

## 4. Treatment

The patient was started on broad spectrum antibiotics and aggressive intravenous fluid resuscitation. Her vital signs stabilized after completing the course of antibiotics and adequate fluid resuscitation; however, her mental status did not improve. She was ultimately intubated for airway protection as well as volume overload from aggressive fluid hydration. She ultimately required a tracheostomy as well as a percutaneous endoscopic gastrostomy tube placement.

The oncology team was consulted for consideration of therapy for ECD-LCH. However, given the patient's poor performance status, she was deemed a poor candidate for interferons/chemotherapy. The benefits and risks of therapy were discussed with the family and their decision was to continue with conservative measures. The patient's acute issues subsequently resolved. She was ultimately transferred to a long term acute care facility due to her poor performance status. Follow-up visits with her primary care physician have shown clinical stability without any acute issues. She is being managed conservatively (without any aggressive therapies) based on her family's wishes.

## 5. Discussion

Histiocytic disorders are exceedingly rare entities which arise from either Langerhans' dendritic cells (antigen-presenting cells) or the monocyte-phagocytic cells (antigen-processing cells) [1]. Such disorders, based on their genesis, are divided into LCH or non-LCH (ECD being the prototype) [2].

Erdheim-Chester disease, first described in 1930 by Jakob Erdheim and William Chester as “lipid granulomatosis,” is a multisystem histiocytosis (non-Langerhans cell type) with a myriad of clinical presentations, distinct radiological findings, and unique immunohistological features [3]. It is a rare variant of non-LCH [3]. The limited reports would suggest it primarily affects individuals between their fifth and seventh decades of life (average 54 years), with a slight predilection towards males [3]. It carries a high mortality (around 60%) in 5 years, which is worse than that found in LCH [3].

ECD is characterized by the symmetrical multifocal osteosclerotic lesions in the diametaphyseal regions of the long bones (especially of the lower extremities), which is virtually pathognomonic for it [4]. More than 650 cases have been described in the literature worldwide [5]. In contrast, the osseous involvement of LCH is typically lytic lesions which can involve any bone of the body [6, 7]. The skeletal or extraskeletal lesions in ECD are the result of infiltration of foamy macrophages (which do not bear the characteristic markers of Langerhans' cells) into these structures. Our patient had evidence of bilateral osteosclerotic lesions of the long bones in her upper and lower extremities. Her maxillae and mandible had evidence of large lytic lesions, which can be seen in both ECD and LCH [8, 9].

The etiology of ECD is not clear, but a multihit hypothesis is favoured. It is hypothesized that either infection or other inciting factors in a genetically predisposed individual can lead to aberration in the regulation of the immune system, which results in initiation and propagation of chemokine induced histiocytic invasion of different sites of the body. Chester proposed histiocyte infiltration to be responsible for multisystem sclerosis and fibrosis, a concept which if manipulated can affect the management and prognosis.

Skeletal involvement is the hallmark of ECD (96%). Pain in bones and/or joints is the most common presenting complaint (around 50%). Almost all of the patients also have extraskeletal involvement (maxillary sinus, heart and great vessels, retroperitoneum, kidneys, lungs, and central nervous system including pituitary and orbit) which determines the supplemental clinical features which the patient exhibits as well as the prognosis of the disease with cardiovascular and/or central nervous system affliction carrying the worst outcomes [10].

ECD is diagnosed on the basis of radiological and immunohistochemical features interpreted in the background of clinical context. One retrospective case series showed that ECD can involve (in order of decreasing frequency) the long bones (95%); maxilla, large vessels, and retroperitoneum (59%); heart (57%); lungs (46%); central nervous system (41%); skin (27%); and orbit (22%) [11, 12].

The bony lesions of ECD can be readily picked up by radiographs and Technetium-99m bone scintigraphs [13]. Immunohistochemical analysis of the bone lesions demonstrates the presence of mononuclear foamy histiocytes, from monocyte-macrophage lineage, which are CD68 positive but negative for CD1a, S100 protein, and Birbeck granules, in contradistinction to the Langerhans' cells [4, 14].

Even rarer in ECD is the coexistence of Langerhans' cells along with the CD1a negative foamy macrophages, on either the same site or different ones [15]. One possible explanation of this additional infiltration of Langerhans' cells in ECD is the chemokines (CCL19, CCL21, and more) released by the foamy macrophages. “Mixed histiocytosis,” a term vaguely used to highlight the presence of features of both LCH and ECD in a single patient, is being increasingly recognized recently. There is evidence to suggest that misguided differentiation of myeloid precursors plays a role in development of ECD and LCH leading to the term “inflammatory myeloid neoplasms” [5, 16]. Fewer than 10 cases had been reported before the work of Hervier et al., who later presented a series of 23 biopsy proven “mixed histiocytosis” cases and also emphasized the importance of BRAF mutations [15, 17–22]. A few striking features noted in mixed histiocytosis which are dissimilar to both ECD and LCH are age, skeletal involvement, and the underlying culprit cells (Table 1).

Age: patients reported in case reports describing mixed histiocytosis are found to have an age between 27 and 43 years. In contrast, ECD is a disease of the elderly with the majority of patients being in the fifth to seventh decades of their life (average 53 years). On the other hand, LCH most commonly afflicts children or younger adults. Skeletal involvement: ECD is characterized by symmetrical osteosclerotic lesions in the diametaphyseal regions of the long bones, especially the femur, tibia, and fibula, whereas asymmetrical osteolytic lesions of the flat bones and the skull are characteristic of LCH. Patients with “mixed histiocytosis” show a combination of osteosclerotic and osteolytic lesions in the bones, at either the same site or a different site.

Underlying culprit cell: in LCH, the causal cell is derived from the Langerhans dendritic cell lineage whereas the monocyte-macrophage system (i.e., foamy macrophages) is the culprit in the pathogenesis of ECD. Patients showing features of both LCH and ECD are thought to have an abnormality in CD 34+ cells, which are progenitors for both cell types described and can be steered in either pathway when cultured in vitro. BRAF (V600E) mutations have been shown to play an important role in the development of these disorders [23].

Most recognized “overlap” cases describe the presence of both disease states simultaneously in the patient, but existence of both of them spaced apart in time has been reported which could represent evolution of LCH into ECD, reflecting the old concept of ECD being “late-stage healing” of Langerhans as was proposed by Pineles et al. [18, 21, 22].

Treatment of ECD is not well established. Interferon alpha, either conventional or pegylated, is the preferred treatment for symptomatic patients, especially those with extraosseous system involvement. In patients with BRAF V600E mutations, vemurafenib (a BRAF inhibitor) has shown promising results [24, 25]. Other options, though controversial, include systemic chemotherapy, glucocorticoids, and radiation therapy [23, 26, 27].

In conclusion, ECD with overlapping features of LCH might represent different spectra of the same disorder. Increasing recognition and reporting of such cases can help clinicians to have a better understanding of the pathophysiology involved, which ultimately can be translated into better management of the therapeutic options. Diagnosis of either of these rare syndromes requires a high index of suspicion, and when suspected these should be evaluated by radiological and immunohistopathological analysis.

## Figures and Tables

**Table 1:** Pertinent differences between the two varieties of histiocytic disorders.

Clinical and immunohistochemical features	Histiocytosis	
	Langerhans' cell	Non-Langerhans' cell
Incidence	Higher	Lower
Mortality	Lower (10%)	Higher (57%)
Culprit cells	Langerhans' cells	Foamy macrophages
Culprit lineage	Langerhans-dendritic cell lineage	Monocyte-macrophage lineage
CD 68	+	+
CD 1a	+	−
S100	+	−
Central nuclear groves	+	−
Birbeck granules	+	−
Age of onset	Children or young adults	The elderly (50s–70s)
Skin involvement	More	Less
Skeletal involvement	Asymmetric osteolytic lesion of skull and flat bones	Symmetric osteosclerotic lesions of long bones
Proliferative index	2–60%	<3%
